# *Drosophila* neuroblasts as a new model for the study of stem cell self-renewal and tumour formation

**DOI:** 10.1042/BSR20140008

**Published:** 2014-07-29

**Authors:** Song Li, Hongyan Wang, Casper Groth

**Affiliations:** *Neuroscience & Behavioral Disorders Programme, Duke-National University of Singapore Graduate Medical School Singapore, 8 College Road, Singapore 169857, Singapore; †NUS Graduate School for Integrative Sciences and Engineering, National University of Singapore, 28 Medical Drive, Singapore 117456, Singapore; ‡Department of Physiology, Yong Loo Lin School of Medicine, National University of Singapore, Singapore 117597, Singapore

**Keywords:** asymmetric division, differentiation, neural stem cell, neuroblast, Notch, self-renewal, tumour formation, ACD, asymmetric cell division, Akt, protein kinase B, *ana2*, *anastral spindle 2*, APC/C, APC/cyclosome, *asl*, *asterless*, Aur-A, Aurora-A, Baz, Bazooka, bHLH-O, basic helix-loop-helix-orange, Brat, brain tumour, CSL, CBF1 (CCAAT-binding factor 1)/suppressor of Hairless/Lag-1, CTF, C-terminal fragment, Ctp, Cut up, Dlg, discs large, DM, dorsomedial, *dMyc*, *Drosophila Myc*, Dpn, Deadpan, EGFR, epidermal growth factor receptor, eIF4E, eukaryotic translation initiation factor 4E, Flfl, Falafel, GMC, ganglion mother cell, GoLoco, Gαi/o-Loco interaction, GSC, glioma stem cell, INP, intermediate neural progenitor, Insc, inscuteable, Klu, Klumpfuss, *l(3)mbt*, *lethal (3) malignant tumour*, Lola, longitudinals lacking, Mira, Miranda, MTOC, microtubule-organizing centre, mTORC2, mammalian target of Rapamycin complex 2, Mud, mushroom body defect, NG2, neuron-glial antigen 2, NICD, Notch intracellular domain, NuMA, nuclear mitotic apparatus, OPG, oligodendrocyte progenitor, Par6, partitioning-defective 6, PCM, pericentriolar material, Pins, partner of inscuteable, PKC, protein kinase C, PntP1, pointed P1, Pon, partner of Numb, PP2A, protein phosphatase 2A, PP4, protein phosphatase 4, Pros, Prospero, Lgl, lethal (2) giant larvae, *Sas-4*, *spindle assembly abnormal 4*, SCD, symmetric cell division, Slimb, Supernumerary limbs, TPR, tetratricopeptide, Zif, zinc-finger

## Abstract

*Drosophila* larval brain stem cells (neuroblasts) have emerged as an important model for the study of stem cell asymmetric division and the mechanisms underlying the transformation of neural stem cells into tumour-forming cancer stem cells. Each *Drosophila* neuroblast divides asymmetrically to produce a larger daughter cell that retains neuroblast identity, and a smaller daughter cell that is committed to undergo differentiation. Neuroblast self-renewal and differentiation are tightly controlled by a set of intrinsic factors that regulate ACD (asymmetric cell division). Any disruption of these two processes may deleteriously affect the delicate balance between neuroblast self-renewal and progenitor cell fate specification and differentiation, causing neuroblast overgrowth and ultimately lead to tumour formation in the fly. In this review, we discuss the mechanisms underlying *Drosophila* neural stem cell self-renewal and differentiation. Furthermore, we highlight emerging evidence in support of the notion that defects in ACD in mammalian systems, which may play significant roles in the series of pathogenic events leading to the development of brain cancers.

## DISRUPTION OF ASYMMETRIC DIVISION AND BRAIN TUMOUR FORMATION

### Apical–basal polarity

**Figure 1:**
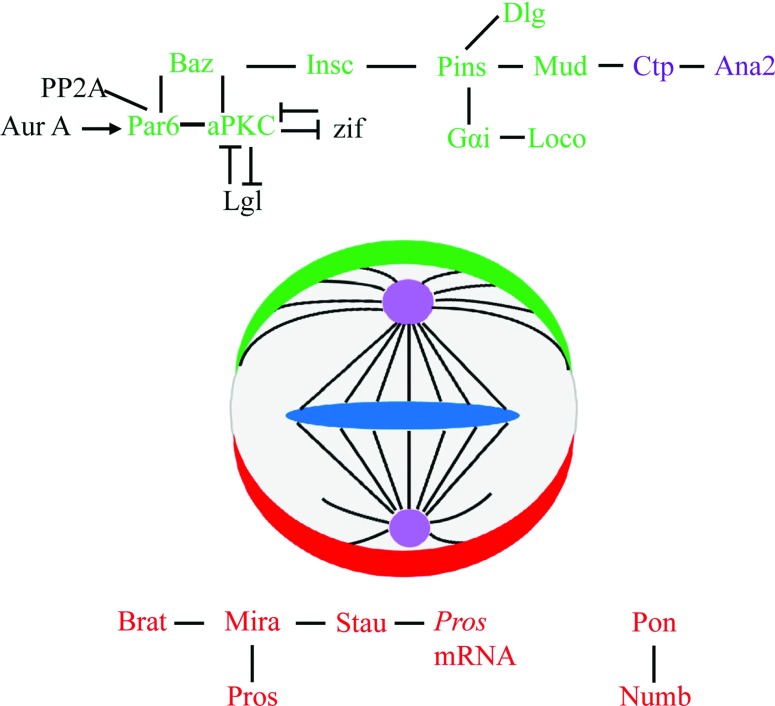
Asymmetric protein localization in the mitotic larval brain neuroblast Apical (in green) and basal (in red) proteins localize asymmetrically in neuroblasts at metaphase. The apical Baz–aPKC–Par6 complex is required for establishing cell polarity, and the Gαi–Pin-s-Loco complex at the apical side regulates the spindle orientation. These two complexes are linked by Insc. The basal protein complexes (Mira–Pros–Brat and Pon–Numb) control differentiation of the daughter GMC independently of each other. Centrosomes and centrosomal proteins are in purple.—indicates the direct interaction; → indicates the positive regulation; ⊤ indicates the negative regulation.

During asymmetric divisions, neuroblasts are polarized to form distinct cortical domains, containing different sets of proteins that are segregated into two different daughter cells by a neural stem cell self-renewal mechanism conserved throughout the embryonic and larval stages. Protein polarity is first established in the prospective embryonic neuroblasts prior to their delamination from the neuroectoderm by the apical localization of the Par complex, consisting of Baz (Bazooka), aPKC (protein kinase C) and Par6 (partitioning-defective 6). All subsequent neuroblast self-renewal divisions, during embryonic and larval stages, recapitulate this initial protein localization asymmetry and use it as the initiating cue for the execution of the ACD (asymmetric cell division) programme by regulating the asymmetric localization of basal proteins (Figure 1; [1–4]). aPKC functions as the effector in this complex and directly phosphorylates the basal proteins Mira (Miranda) and Numb to restrict their asymmetric localization [5–7]. In *aPKC* mutants, there are fewer neuroblasts per brain lobe compared with wild-type and the neuroblasts stop dividing prematurely to generate smaller lineages [2,8]. Conversely, the activation of aPKC at the entire cell cortex of neuroblasts results in asymmetric division defects, leading to a dramatic increase in the number of neuroblasts [8]. The asymmetric localization of the basal protein is regulated by apical proteins through cortical tumour suppressor proteins Lgl (lethal (2) giant larvae) and Dlg (discs large) [9,10]. Lgl associates with aPKC and Par6 and is subject to aPKC-dependent phosphorylation and inactivation at the apical cortex. This leads to its disassociation from membranes and the actin cytoskeleton, thus restricting the localization of Mir to the basal cortex [11]. Lgl also acts as an inhibitor of aPKC in neuroblasts, restricting aPKC apical localization [8]. The Par complex is also regulated by Aur-A (Aurora-A) in neuroblasts. At the onset of mitosis, Aur-A-mediated phosphorylation of Par6 releases Lgl from the complex, thus enabling Baz to form a tripartite complex with aPKC and Par6, which facilitates the phosphorylation of Numb by aPKC [6,7,11]. The transcription factor Zif (zinc-finger protein) binds directly to the promoter region of *aPKC* gene and acts to repress the expression of *aPKC* [12]. Interestingly, aPKC-dependent phosphorylation of Zif, leads to its exclusion from the nucleus and makes it functionally inactive [12]. Therefore the mutual interplay between Zif and aPKC is critical for proper activity of aPKC during neuroblast asymmetric division. The components of the Par complex are evolutionarily highly conserved and mutations in genes encoding Par complex proteins are associated with hyperproliferation, tumour formation and increased metastasis in humans [13]

Basally localized proteins are segregated into the GMC (ganglion mother cell) during neuroblast asymmetric division and are important for GMCs to undergo differentiation pathway. The proliferative potential of the GMC is limited to a single division, which generates two post-mitotic neurons or glial cells with distinct cell fates [14]. To date, three proteins, Numb, Pros (Prospero) and Brat (Brain Tumour) have been identified to specify the GMC fate in *Drosophila* neuroblasts (Figure 1; [15–18]. The localization of both Pros and Brat at the basal cortex is dependent on their interaction with the adaptor protein Mira [19–21]. In the absence of Mira, Pros and Brat are localized throughout the cytoplasm of neuroblasts at metaphase, and are segregated into both the GMC and the neuroblast during ACD. Pros is a member of the ProX1 family of transcription factors, which translocates to the nucleus to repress the genes for neuroblast self-renewal and promote the genes for GMC differentiation, following its segregation into the GMC [22]. Brat acts redundantly with Pros to control the GMC fate in *Drosophila* embryos, whereas in larval brains it functions as a tumour suppressor to inhibit neuroblast self-renewal and promote neuronal differentiation [20,21,23]. A second basal complex is composed of Numb and the adaptor protein Pon (partner of Numb) [24]. Pon facilitates the polarized localization and segregation of Numb during neuroblast asymmetric division [24,25]. Numb inhibits the Notch signalling pathway by binding to the NICD (Notch Intracellular Domain) and promoting the endocytosis of the Notch receptor, thereby reducing the signalling competent Notch pool available at the cell surface (see below; [26]). Following GMC division, two neurons or glia with different fates are generated, and Numb is asymmetrically segregated into one neuronal sibling [26]. In the absence of Numb, the GMC produces two daughter cells with the same identity [26]. Moreover, Numb functions as a tumour suppressor in larval brains and supernumerary neuroblasts form in *numb*-deficient neuroblasts [21,27,28].

Interestingly, when larval brain tissue, mutant for genes encoding ACD regulators (e.g., *aur-A*, *lgl* and *mira*) or cell fate determinants (*brat*, *numb* and *pros*), is implanted into the abdomen of adult wild-type hosts, tumour formation ensue [29]. The implanted cells in the tumours become immortal and can proliferate almost indefinitely [29]. These data suggest a link between the disruption of asymmetric division and tumourigenesis in *Drosophila* larval brain tissue.

### Spindle orientation

The alignment of the apical–basal polarity axis with the mitotic spindle is referred to as spindle orientation. It is essential to position the cleavage furrow during cytokinesis to ensure the exclusive segregation of apical or basal proteins into different daughter cells. The apical protein Insc (inscuteable) is a key regulator of neuroblast mitotic spindle orientation in *Drosophila* [30]. Insc is recruited by the Par complex during neuroblast mitosis and binds co-dependently to the Pins (partner of inscuteable) complex to secure their asymmetric localization at the apical cortex (Figure 1; [31,32]). Pins contains multiple mushroom body defect TPR (tetratricopeptide) motifs and three GoLoco (Gαi/o-Loco interaction) repeats at its C-terminus, which interact to form an inactive protein [32,33]. Activation of Pins is achieved by the binding of the heterotrimeric G protein Gαi to the GoLoco repeats, resulting in a conformational change and activation of the Pins protein [33]. Active Pins can interact with Mud (mushroom body defect) directly through TPR domains and recruit Mud to the apical cortex [33,34]. *Drosophila* Mud is an orthologue of the mammalian NuMA (nuclear mitotic apparatus) protein, which is critical for aster formation and stability of microtubules in mammals [35]. Mud localizes at both the apical side and centrosomal regions in neuroblasts, and is important for proper spindle orientation [34,36,^37^]. Another pathway implicated in regulating neuroblast spindle orientation is composed of Pins, Gαi and the tumour suppressor Dlg [38,39]. Dlg binds to the Pins linker domain, which connects the Pins TPR and GoLoco domains [40]. A plus-end-directed microtubule motor protein Khc73 (Kinesin heavy chain 73) interacts with Dlg to control spindle orientation through regulating Pins cortical polarity in neuroblasts [39]. Therefore Pins regulates neuroblast spindle orientation by interacting with downstream proteins Mud or Dlg.

Centrosomes serve as the main MTOC (microtubule-organizing centre) of the animal cell and is an organelle that plays a key role during cell division. A centrosome consists of two centrioles surrounded by an amorphous mass of proteins called PCM (pericentriolar material) [41]. What is the correlation between mother versus daughter centrosome segregation and asymmetric cell fate in stem cells? In *Drosophila* male germline stem cells, the mother centrosome is retained by the stem cells, whereas the daughter centrosome is segregated into the differentiating gonial cell following division [42]. Interestingly, the two centrosomes also behave differently during asymmetric division of neuroblasts. After centriole duplication at interphase, the two centrioles split and are functionally different [43,44]. The daughter centriole is active and retains PCM, thus becoming a MTOC of the cell [43–45]. This centrosome remains at the apical side and is segregated into the new-born neuroblast after cell division. Conversely, the mother centriole loses the PCM soon after centriole duplication and separation and moves to the basal side of the neuroblast, and is segregated into the cell fate-restricted daughter cell [43–45]. Centrobin is an essential centriolar protein required for centrioles in order to retain PCM and organize the interphase aster in neuroblasts in a Polo phosphorylation-dependent manner [46]. In contrast, the PLP (pericentrin-like protein) that is enriched on the inactive interphase centrosome, blocks the recruitment of Polo to the centrosomes and in turn inhibits the activity of interphase centrosomes [47]. In the absence of this centrosome asymmetry, the site of GMC budding is not efficiently maintained from one cycle to the next [46].

Loss of either centrioles or PCM would compromise the functions of centrosomes in the neuroblasts; hence, in mutants that lack centrioles or PCM, spindle misorientation phenotypes are often observed. For example, in *centrosomin* (*cnn*) mutant neuroblasts, microtubule defects and spindle misorientation phenotypes occur, and this may lead to a significant increase in the number of neuroblasts because of missegregation of cell fate determinants [48,49]. Consistently, mutants of several centriolar proteins, including *asl* (*asterless*), *ana2 (anastral spindle 2*) and *Sas-4* (*spindle assembly abnormal 4*), display spindle misorientation and/or neuroblast overgrowth phenotypes [50–52]. *ana2* mutant larval neuroblasts lack centrosomes and display severe spindle orientation defects [52]. Ana2 directly interacts and anchors Ctp (Cut up), a cytoplasmic dynein light chain, at the centrosomes [52]. Ana2 and Ctp bind to Mud, and are important for the Pins–Mud interaction by controlling the centrosomal and apical localization of Mud [52]. Therefore Ana2 is important for neuroblast spindle orientation by regulating microtubule-mediated spindle–cortex interactions [52].

Failure in proper mitotic spindle orientation may result in missegregation of asymmetrically localized proteins and lead to neuroblast overgrowth [34,36,52]. In *mud* or *ana2* mutants, neuroblast symmetric divisions occur most probably because of orthogonal divisions that result from severe defects in the orientation of the spindle. In this abnormal form of division, apical and basal proteins are found equally segregated into the two daughter cells, making the two daughter cells both capable to self-renew [34,36]. Consistently, the mutant brain tissues of *pins*, *mud* or genes encoding centrosomal proteins, such as *cnn*, *asl*, *sas-4* and *ana2*, can induce the formation of tumours in allograft assays [50–53].

### Cell-cycle regulators and post-translational modifications in neuroblasts

The cell polarity in neuroblast is determined in the early interphase; however, the polarized proteins begin to localize asymmetrically at the prophase during the cell cycle, suggesting the roles of cell-cycle regulators in monitoring neuroblast asymmetric division. Two cell-cycle-related kinases have been well characterized in *Drosophila* neuroblasts: Aur-A and Polo kinases [25,28,48,54,55]. Loss of Aur-A or Polo leads to the formation of ectopic neuroblasts in larval brains [25,28]. In *aur-A* mutant larval neuroblasts, aPKC is delocalized to the entire cortex and the asymmetric distribution of Numb is largely disturbed [28,48]. Moreover, Aur-A directly phosphorylates Par6 at interphase, which facilities the formation of the Baz/Par6/aPKC complex and is essential for aPKC-mediated regulation of Numb [7]. Aur-A phosphorylates a specific site within the evolutionarily highly conserved Pins linker domain of the Pins protein to orient the mitotic spindle [40]. Aur-A may also regulate mitotic spindle orientation by controlling the apical localization of Mud, as Mud is distributed throughout the cell cortex in *aur-A* mutant neuroblasts [28]. Polo kinase controls Numb asymmetry by phosphorylating Pon, the binding partner of Pon, leading to the polarized localization of Pon and Numb in neuroblasts [25]. In addition, Polo is required for the proper localization of aPKC and correct spindle orientation in neuroblasts, suggesting that Polo inhibits neuroblast self-renewal through regulating the localization/activity of Numb and the orientation of mitotic spindles [25].

Given the critical roles that protein kinases (Aur-A, Polo, aPKC) play in neuroblast self-renewal and differentiation, it is likely that protein phosphatases may regulate neuroblast homoeostasis by counteracting the activities of these kinases. Surprisingly, PP2A (protein phosphatase 2A) loss-of-function mutants result in defects in asymmetric division and form supernumerary neuroblasts in *Drosophila* larval brains [56,57]. PP2A is a conserved serine/threonine phosphatase that functions as a heterotrimeric complex comprising a catalytic C subunit [Mts (microtubule star)], a scaffolding A subunit (PP2A-29B) and one of the variable regulatory B subunits; Twins (Tws), Widerborst (Wdb), B56-1 or PR-72. The A subunit of PP2A bridges the catalytic subunit and the B subunits, which provide the substrate specificity [58]. PP2A inhibits neuroblast self-renewal by functioning upstream of Polo [57]. On the other hand, PP2A also negatively regulates aPKC activity by associating with Par6 and dephosphorylating Par6 in order to counteract Aur-A-mediated phosphorylation of Par6 [59]. In addition, PP2A interacts with Baz via its catalytic subunit (Mts) and dephosphorylates Baz at the conserved serine 1085, which is important for the proper cell polarity in embryonic neuroblasts [60]. Moreover, PP2A can directly dephosphorylate Numb to facilitate the repression of neuroblast self-renewal [61].

Another protein phosphatase reported to mediate neuroblast ACD is PP4 (protein phosphatase 4). Loss of Flfl (Falafel), a regulatory subunit of PP4, leads to a mis-localization phenotype of the basal protein complex including Mira and its cargo proteins Pros, Brat and Stau (Staufen) in metaphase/anaphase neuroblasts [62]. Flfl interacts with Mira, indicating that Flfl might target PP4 to the Mira complex for its proper association/asymmetric localization during neuroblast asymmetric division [62].

In addition to protein phosphorylation, the E3 ligase APC/C (APC/cyclosome) complex has been shown to regulate asymmetric localization of Mira and its cargo proteins during neuroblast asymmetric division [63]. Mira is ubiquitinated through its C-terminal domain, which contains an APC/C destruction motif [63]. The ubiquitination of Mira is important for its polarized localization during neuroblast asymmetric division, and attenuation of APC/C activity in neuroblasts displayed a Mira-delocalization phenotype similar to *mira* mutants in which the C-terminal Mira is dysfunctional [63]. Interestingly, SCF^Slimb^, an evolutionarily conserved E3 ubiquitin ligase complex, consisting of Cul1 (Cullin1), SkpA, Roc1a and the F-box protein Slimb (Supernumerary limbs), regulates asymmetric division of neuroblasts and inhibits the formation of ectopic neuroblasts [64]. Two direct targets of SCF^Slimb^, SAK (Sak kinase) and Akt (protein kinase B) function downstream of the SCF^Slimb^ complex during neuroblast self-renewal [64].

### Neuroblast lineages in *Drosophila* central brains

**Figure 2:**
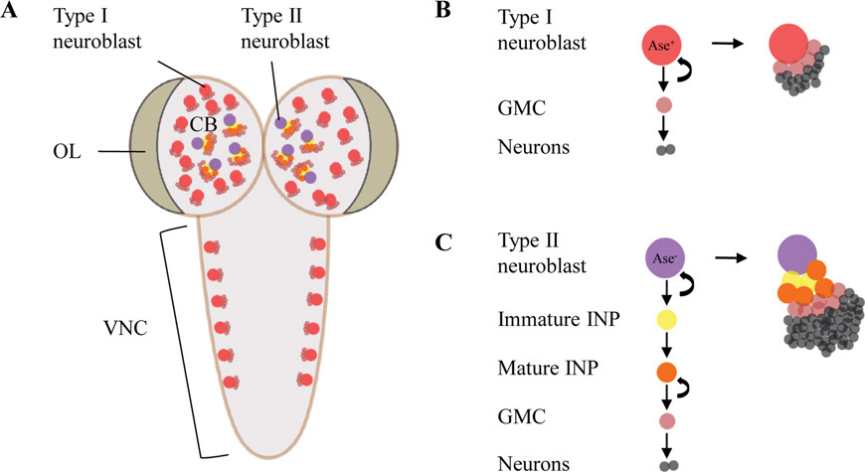
Neuroblast lineages in the *Drosophila* larval brain (**A**) A dorsal view of *Drosophila* third instar larval brain which contains three main neurogenic regions: central brain (CB), optic lobe (OL) and ventral nerve cord (VNC). Type I neuroblasts (in red) and type II neuroblasts (in purple) are located at CB. (**B**) Type I neuroblasts divide asymmetrically to self-renew and produce a GMC (in light red). GMC divides one more time to generate neurons (in grey). (**C**) Type II neuroblasts divide unequally to generate a self-renewing neuroblast and an immature intermediate neural progenitor (INP, in yellow). After maturation, the INP (in orange) divides asymmetrically to self-renew and generate a GMC.

The larval central brain contains approximately 100 neuroblasts in each brain hemisphere, which are divided into type I and II lineages, based on differences in gene expression and progeny types (Figure 2; [27,65,66]). Both types of neuroblasts divide asymmetrically to generate two distinct daughter cells during larval development. Type I neuroblasts constitute the predominant population, and express *asense* (*ase*), a proneural gene that encodes a nuclear transcription factor [67], and cytoplasmic or basally localized Pros [16]. A type I neuroblast undergoes asymmetric division to generate a self-renewing neural stem cell and a GMC, which divide once more to produce two postmitotic neurons or glia. Type II neuroblasts constitute a minor population with only 8 per hemisphere, which do not express Ase and Pros, and divide asymmetrically to generate a neuroblast and a small daughter cell, termed an INP (intermediate neural progenitor). The new-born INP is present in an immature, non-proliferative state characterized by the lack of Ase and Pros expression. The immature INP quickly transits to become an Ase-positive mature INP, in a process dependent on Brat and Numb activity. The mature INP is competent to undergo several rounds of asymmetric divisions, each time generating a self-renewing INP and a GMC [27,65,66]. Therefore, a type II neuroblast generates a larger lineage than a type I neuroblast in *Drosophila* larval brains due to the restrictive self-renewal capability of the INPs. Among eight type II neuroblast, six of them locate at the DM (dorsomedial) larval brain lobe named DM1–DM6, and the other two locate at more lateral positions [66].

The development of type II neuroblast lineages in *Drosophila* mimics that of mammalian neural stem cells, which contain transit-amplifying cells [68,69]. Therefore *Drosophila* type II neuroblast lineages have emerged as an attractive model system to study the fundamental molecular network controlling the cell fate specification and proliferation of neural stem cell lineage progenitors. Brat and the Notch antagonist Numb, function cooperatively to ensure that immature INPs undergo maturation and commit to the INP fate [27]. Several transcription factors have been identified to be bona-fide regulators of neuroblast lineage identity. *Earmuff* (*erm*), which is an orthologue of the vertebrate *Fezf* (*Forebrain embryonic zinc-finger family*) genes, encoding transcription factors, functions specifically in INPs [70]. Loss of Erm in type II neuroblast lineages leads to a failure in the ability of the INPs to maintain their cell fate identity, allowing some INPs to dedifferentiate back into a neuroblast state [70]. Erm restricts the potential of INPs by attenuating their response to self-renewal factors [71]. The Brahma remodelling complex, together with histone deacetylase 3, physically associates with Erm to suppress INP dedifferentiation back into neuroblasts [72]. Osa, a subunit of the Brahma chromatin-remodelling complex, induces the expression of *hamlet* (*ham*), a member of the *Prdm* gene family, in INPs to limit the proliferation of INPs [73]. PntP1 (pointed P1), which belongs to the Ets (E26 transformation-specific) transcription factor family, is a key player in the control of type II neuroblast lineage identity. It suppresses Ase expression in type II neuroblasts and promotes the generation of INPs, as loss of PntP activity in type II neuroblasts leads to the reduction or elimination of INPs [74]. The zinc-finger transcription factor, Klu (Klumpfuss), acts as a neuroblast self-renewal factor, and its expression distinguishes a type II neuroblast from an INP in larval brains. Misexpression of Klu triggers immature INPs to revert to type II neuroblasts [75,76]. A number of transcription factors regulate the expression of neuronal identity factors to maintain neuroblast homoeostasis. For example, the Snail family protein Worniu maintains neuroblast self-renewal by preventing Elav-induced premature neuronal differentiation [77], and Midlife crisis, a conserved zinc-finger protein, maintains Pros and Elav in post-mitotic neurons to inhibit neuronal dedifferentiation [78]. Other mechanisms that prevent dedifferentiation of INPs, GMCs or neurons will be of great interest for neural stem cell studies.

## THE NOTCH SIGNALING PATHWAY AND NEURAL STEM CELL SELF-RENEWAL

### The Notch signalling pathway

**Figure 3:**
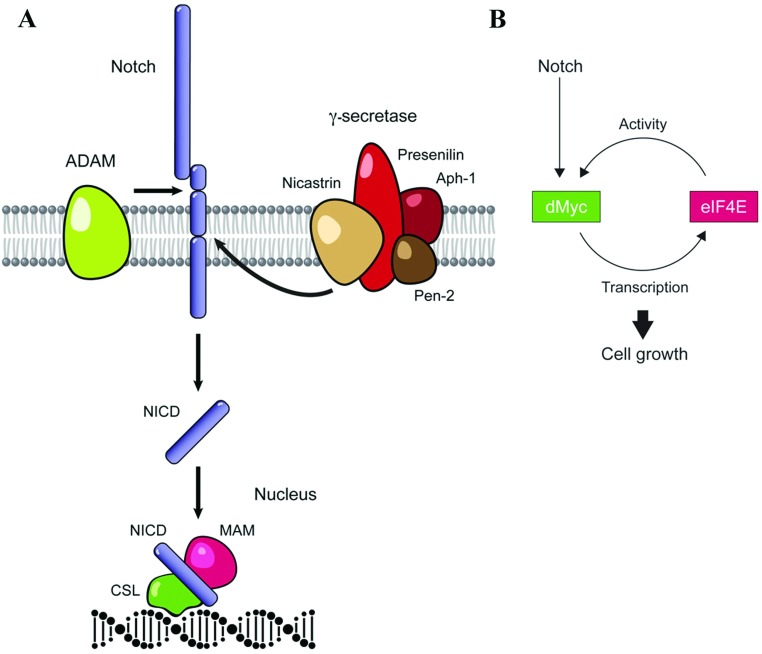
The Notch signalling pathway and dMyc-mediated cell growth (**A**) The Notch receptor is processed sequentially by three enzymatic activities. In the Golgi compartment, Notch is cleaved by furin to produce two covalently linked fragments, which form the mature protein. After reaching the cell surface, Notch is activated and processed following the interaction with a ligand and an ADAM protease to release its ectodomain into the extracellular matrix. This shedding event renders the truncated membrane-bound Notch remnant accessible to the action of the γ-secretase complex, which cuts Notch within the confines of the lipid bilayer to release the Notch intracellular domain, NICD. This gene regulatory fragment then moves to the nucleus where it interacts with various co-activators, including CSL and MAM to regulate target gene expression. (**B**) The growth regulator dMyc is a target of the Notch pathway. One of the targets regulated by dMyc is the translation initiation factor eIF4E, which acts as a cell growth mediator. The dMyc/eIF4E regulatory loop functions to control cell growth and cell fate choices in type II neuroblast lineages (based on [100]).

The Notch signalling pathway plays essential roles in many cellular processes, including cell fate specification, cell proliferation and cell death events in order to regulate the establishment and maintenance of cell types and tissues during embryonic development and adult tissue homoeostasis (reviewed in [79]). Notch-dependent signal transduction ensures a local and highly specific signal exchange between neighbouring cells engaged in cell–cell interactions. The interaction between ligand- and Notch-expressing cells directs the transmission of a signal to the nucleus of the Notch-expressing cell to regulate target gene expression without the involvement of any enzymatic amplification steps. This feature is achieved by the stepwise proteolytic processing of the Notch receptor, ultimately leading to the release of a nucleus-targeted gene-regulatory intracellular domain, termed the NICD (reviewed in [79]). The maturation and activation of Notch is orchestrated by a series of enzymatic cleavages found near its TM (transmembrane) domain (Figure 3). Notch is synthesized as a precursor protein of approximately 300 kDa, which is predominantly cleaved by furin-like convertases in the *trans*-Golgi compartment to form NTF (N-terminal fragment) and CTF (C-terminal fragment), respectively. The two fragments are subsequently linked by non-covalent bonding to form the mature Notch heterodimer [80,81]. Following protein maturation, Notch is trafficked to the cell surface and activated as a consequence of conformational changes facilitated by specific binding to ligands of the DSL family, which includes Delta and Serrate/Jagged in *Drosophila* and mammals as well as LAG-2 in *Caenorhabditis elegans*. Ligand binding then triggers a second cleavage event in the extracellular region of the Notch CTF mediated by ADAM metalloproteases, which leads to the shedding of the extracellular domain [82–84]. The remaining membrane-anchored Notch fragment is subsequently cleaved by the intramembrane aspartyl protease γ-secretase to release the nuclear-bound NICD fragment (Figure 3; [85–87]). In addition to the control of Notch processing, Notch signalling is also regulated by post-translational modifications, control of protein trafficking and degradation through the secretory pathway and endocytic and lysosomal compartments (reviewed in [79]).

### The role of Notch in asymmetric division and stem cell self-renewal

Notch-mediated binary pattering processes are essential in regulating cell fate specification events associated with neuroblast self-renewal and differentiation in the developing brain (reviewed in [88]). During larval neurogenesis, type I and II neuroblasts are to a variable degree dependent on the Notch pathway to maintain neuroblast self-renewal [27,28,89]. A common regulator between the two distinct stem cell populations is the Notch pathway inhibitor Numb [27,28,48]. This protein plays a pivotal role by enabling binary cell fate specification events to occur as a result of its asymmetric sequestration in post-mitotic neurons [28,88,90], How Numb exert its inhibitory function on Notch signalling remains poorly understood. However, a number of studies have implicated Numb as an endocytic regulator of the Notch signalling enhancer Sanpodo [91]. Numb interacts with both Sanpodo and the endocytic sorting machinery protein α-adaptin to promote Sanpodo endocytosis [92–94]. This leads to a reduction of Sanpodo at the cell surface and, consequently, the down-regulation of the Notch pathway in the Numb-positive daughter cell. Conversely, the Numb-negative cell has high Sanpodo levels and retains its neuroblast identity [95,96].

If Numb activity is abnormal in the larval brain, then the balance between neuroblast self-renewal and differentiation will be compromised. As a consequence, Notch signalling becomes dysregulated in neuroblasts, which leads to cell fate transformation and cause daughter cells to adopt a neuroblast-like identity at the expense of neuronal differentiation. This leads to unrestrained proliferation of self-renewing neuroblast-like cells, and tumour formation ensues in the larval brain [27–29]. Importantly, the two distinct neuroblast lineages show important differences in their responsiveness towards Notch signalling. In type II neuroblasts, an increase of activated Notch or NICD activity, or the lack of Numb protein in *numb* mutant tissue, lead to neuroblast hyperplasia and a concomitant reduction in the number of differentiating cells, while Notch loss-of-function or down-regulation of Notch signalling using RNAi-mediated inhibition of Notch expression leads to the elimination of type II neuroblasts [27,28,97–99]. The same Notch hyperactivity lesions in type I neuroblasts exhibit significantly lower penetrance of the overgrowth phenotype, suggesting that Notch signalling may be of less importance in controlling proliferation of type I neuroblasts. Alternatively, Notch in conjunction with other proteins may act redundantly to secure robust control of type I neuroblast homoeostasis (see below; [89]). As the main difference between the two neuroblast lineages lies in the presence on an extra cellular amplification step in the form of INP cells in type II neuroblast lineages, this strongly suggests that Notch activity and the regulatory function of Numb may prevent INP populations from resuming neuroblast identity [27,100]. So control of protein polarity and the fidelity of the ACD machinery emerge as important control mechanisms to ensure neuroblast homoeostasis and the prevention of tumour formation in *Drosophila*.

While Notch-dependent control of neuroblast homoeostasis is predominantly mediated via NICD and its interaction with Su(H) to regulate target gene expression [27,70,100], non-canonical Notch signalling also plays a role in maintaining the neuroblast pool. Notch can interact with the Parkinson's disease and cancer associated PINK1 protein to regulate mitochondrial function via mTORC2 (mammalian target of Rapamycin complex 2/AKT signalling [101,102]. The canonical and non-canonical Notch signalling pathways act in conjunction to secure neuroblast homoeostasis. If the activity of the Notch/PINK1/mTORC2/AKT signalling axis is reduced, neuroblast maintenance is compromised [101]. Interestingly, maintenance of *Drosophila* and human neural cancer stem cells exhibit a preferential dependency on the non-canonical Notch pathway compared with normal stem cells [101].

### The role of Notch target genes in neuroblast homoeostasis and beyond

Notch signalling transduction relies on the nuclear translocation of the gene regulatory NICD fragment to control target gene expression. In the absence of NICD, most Notch target genes are maintained in an inactive state because of their interaction with a transcriptional repressor complex, encompassing the CSL [CBF1 (CCAAT-binding factor 1)/suppressor of Hairless/Lag-1] transcription factor Suppressor of Hairless, Su(H), and various corepressors. In the presence of NICD, the corepressors associated with Su(H) binding are displaced with Mastermind and other gene coactivators, leading to activation and transcription of Notch target genes (reviewed in [79]). Given the essential role of Notch signalling in controlling numerous cell fate specification and differentiation events during development and adulthood, we know surprisingly little about the genes targeted by Notch to regulate these processes [88]. Among the identified Notch targets in *Drosophila* are a number of genes encoding bHLH-O (basic helix-loop-helix-orange) transcription factors, implicated in controlling neurogenesis by acting as neural differentiation repressors, including neuroblast marker Dpn (Deadpan) and members of the Enhancer of split complex, E(spl) [89,103].

Hyperactivity of Dpn in type II neuroblasts mimics the Notch pathway overexpression phenotype with accompanying overgrowth of neuroblast-like cells and tumour formation [103]. However, loss of *dpn* activity does not recapitulate the neuronal hypoplasia seen in Notch pathway mutants [74,103]. This conundrum seems to indicate that Dpn may act redundantly with other proteins to regulate type II neuroblast homoeostasis. Indeed, recent reports suggest that both Dpn and members of the E(spl) complex may act synergistically to retain the self-renewing status of type II neuroblasts [89,103].

Recently, *hey* has been identified as a putative Notch target gene and is broadly expressed in Notch-responsive newly born postmitotic sibling neurons during embryonic and larval development [104]. Contrary to most bHLH-O transcription factors identified so far, Hey is not a repressor of neuronal identify and is accordingly not expressed in neuroblasts, but rather exerts its function by promoting the Notch-dependent fate decision in one of the asymmetrically dividing and emerging sibling neurons. This further emphasizes the importance of the link between protein polarity and ACD in neurogenesis.

## MAMMALIAN STUDIES OF ASYMMETRIC DIVISION AND HUMAN CANCERS

Significant progress has been made to identify and elucidate the core molecular machinery behind the homoeostatic balance regulating *Drosophila* neuroblast self-renewal and differentiation and the genetic mutations leading to tumourigenesis. Although the components of the ACD machinery, polarity proteins and polarized cell fate determinants are highly conserved in evolution, it remains largely unknown how significant a role ACD plays in human tumour formation [105,106]. However, a number of recent studies have elucidated various aspects of ACD and tumourigenesis. For example, Izumi and Kaneko [107] reported on the incidence of ACD versus SCD (symmetric cell division) in neuroblastoma cell lines. Neuroblastomas are common childhood solid tumours with variable treatment prognosis. Among the abnormal chromosomal and genetic features associated with this class of tumours is the amplification of the proto-oncogene *MYCN*, a member of the *MYC* gene family, which encode transcription factors involved in the regulation of cell proliferation and growth [108]. The copy number of *MYCN* has been shown to be a good predictor of phenotypic aggressiveness and clinical outcome [109]. In neuroblastoma cell lines with normal *MYCN* copy number the presence of ACD is significantly higher than in cell lines with *MYCN* amplification. Moreover, overexpression of *MYCN* in cells with normal *MYCN* copy number shows an increase of SCD. Conversely, RNAi-mediated inhibition of *MYCN* expression in *MYCN*-amplified neuroblastoma cell lines causes a further increase in the proportion of cells undergoing ACD [107]. These data suggest that MYCN may act as a regulator of the ACD machinery.

Pathogenic disruption of the homoeostatic balance of neural stem cell self-renewal and maintenance is bound to involve the dysregulation of numerous genes to generate the tumourigenic phenotype. For example, CD133, a stem cell marker known to be hyperactivated in cancer stem cells [110], was found to be exclusively expressed in *MYCN*-amplified neuroblastoma cell lines, where it promotes the survival and proliferation of the tumour cells [107], and was shown to asymmetrically co-segregate with Numb during ACD in glioma stem cells [111].

Mechanistic insight into the role of MYC proteins in neural stem cell homoeostasis has been gained from studies of the single *MYC* gene present in the *Drosophila* genome. *dMyc* (*Drosophila Myc*) encodes a transcription factor [112], which regulates cell and organismal growth [113] by controlling the expression of genes involved in ribosome biogenesis [114], and RNA and protein synthesis [115], including transcription and translation factors [100,116–118]. Interestingly, recent findings suggest that the regulation of growth rate and cell size may be a contributing factor in the control of neuroblast cell identity and maintenance [100].

During larval neuroblast cell divisions, the tumour suppressor Brat is asymmetrically segregated into one of the two daughter cells, where it acts as a post-transcriptional regulator of dMyc to inhibit cell growth and proliferation of the cell destined to become the GMC [15]. However, overexpression of *dMyc* alone is insufficient to induce neuroblast overgrowth [15]. Hyperactivation of the Notch pathway in type II neuroblasts, leads to up-regulation of dMyc, and together with the eIF4E (eukaryotic translation initiation factor 4E), their combined action appear to be sufficient to cause the dedifferentiation of immature INPs to type II neuroblast-like progenitor cells (Figure 3; [100]). Ectopic neuroblasts show a significant higher growth rate than normal neuroblasts, while at the same time their ability to sustain growth is more dependent on *eIF4E* function [100]. This raises the hope that differences in the properties of the gene regulatory network underlying normal and cancer neuroblasts may be exploited therapeutically [100].

A switch from ACD to SCD has also been described to occur in malignant gliomas. Using single-cell-based lineage tracing of tumour cells obtained from human clinical specimens, it was recently established that GSCs (glioma stem cells) employ both symmetric and asymmetric modes of propagation to self-renew and generate tumours [111]. Symmetric divisions was found to be the predominant form of GSC propagation, while a lesser portion of the GSCs undergo ACD and may account for a minor part of the cellular heterogeneity in the tumour [111]. Interestingly, in normal brain tissue, OPGs (oligodendrocyte progenitors) undergo ACD as a prominent mode of cell division to self-renew, while tumourigenic OPGs found in human oligodendrogliomas show reduced ACD. This switch in cell division mode is associated with dysfunctional regulators of ACD, including Trim32, a mammalian protein related to *Drosophila* Brat [119]. Moreover, during mitosis of OPGs, the lineage marker NG2 (neuron-glial antigen 2) becomes asymmetrically inherited to one of the daughter cells, which continues to have progenitor identify and self-renewal capacity due to its ability to promote asymmetric localization of EGFR (epidermal growth factor receptor), a transducer of proliferation and self-renewal signals, while the NG2-negative daughter undergo differentiation. Importantly, in a mouse model, the degree of NG2 asymmetry has been causally linked to its tumour-initiating potential [119].

The optic lobe of *Drosophila* is a brain centre involved in the processing of visual information, and its development has provided valuable insights into the molecular mechanisms underlying the switch between SCD and ACD. Neuroepithelial cells in the larval optic lobes undergo symmetric divisions to expand the neural stem cell progenitor pool, whereas the neuroepithelial cell-derived neuroblasts undergo ACDs to produce the differentiated neurons of the visual processing center [68,120–122]. The transition between the two modes of division is dependent on the Notch signalling pathway. A key function of Notch activity is to maintain the neuroepithelial cell state and prevent the transition and development of the optic lobe neuroblasts, as absence of Notch activity in neuroepithelial cells leads to a switch from SCD to ACD and the extrusion of neuroblasts from the neuroepithelium, causing premature neurogenesis [121,123]. Cross-talk between the Notch, EGFR, JAK/STAT and Fat-Hippo pathways are needed for the precise timing and execution of the cell fate specification programme underlying the neuroepithelial to neuroblast cell transition [121,123–128]. The integration of the output from these pathways promote the transient expression of the proneural gene *lethal of scute* (*l’sc*), which propagates through the neuroepithelium and initiate the transformation of neuroepithelial cells into neuroblasts by regulating the switch from SCD to ACD [68]. This switch also requires signals emanating from a subpopulation of optic lobe-associated cortex glial cells. The microRNA *mir-8* from these glial cells promotes the expression of Spitz, an EGFR ligand, which binds and activates the EGFR receptor, leading to the promotion of neuroepitheilial cell proliferation and neuroblast formation [129].

Tumours can arise in optic lobes due to dysregulated signalling in neuroepithelial cells or dedifferentiation of neurons. Derepression of the Hippo pathway in *l(3)mbt* [*lethal (3) malignant tumour*] mutant neuroepithelial cells promotes brain tumour formation [130]. After neurons are born, it is important to prevent them from dedifferentiating back into dividing neuroblasts. A recent finding showed that a BTB-Zn finger transcription factor Lola (longitudinals lacking) represses neural stem cell genes and cell-cycle genes in post-mitotic neurons [131]. In *lola* mutants, newly born post-mitotic neurons can dedifferentiate and assume neural stem cell-like properties, leading to tumour formation in the optic lobe of the adult brain [131].

## CONCLUSIONS AND FUTURE PERSPECTIVES

Much of our basic knowledge about the molecular machinery controlling neural stem cell homoeostasis and ACD has been gained from *Drosophila* neuroblasts. With the identification of the type II neuroblast lineages in *Drosophila* that are analogous to mammalian neural stem cell lineages, we anticipate that unravelling the molecular machinery controlling the self-renewal and differentiation of type II neuroblasts and INPs will provide valuable insights relevant to our understanding of mammalian neural stem cell biology and the pathological processes involved in neural stem cell-dependent brain tumour formation and disease progression.

Although the core components and the regulatory machinery needed for the segregation of asymmetric cell fate determinants in the neuroblast have been determined, it remains largely unresolved how their asymmetry translates into different cell fate acquisitions by their progeny. In type II neuroblast lineages, the INPs are subject to progressive restrictions in their developmental potential, thus allowing them to undergo a limited number of cell division cycles to generate GMCs that give rise to post-mitotic neurons without disrupting the homeostasis of the neuroblast pool. The molecular gatekeepers and mechanisms upholding the developmental barrier, which under normal conditions prevent immature INPs from dedifferentiating and assuming neural stem cell-like properties, are only now slowly beginning to be identified. This area of research deserves special focus, as the developmental switch from neural stem cell to precursor cell identity is a likely key event in the pathogenic cascade responsible for the transformation of neural stem cells to tumourigenic cancer stem cells.

The brain consists of a vast variety of neuronal cell types. How this cellular diversity is generated is one of the greatest unsolved mysteries found in the intersection between stem cell and developmental biology. It is clear that the Notch signalling pathway plays a pivotal role in this process. How Notch signalling asymmetries integrate into the spatiotemporal molecular code underlying neuronal cell diversity promise to be a fruitful avenue of *Drosophila* research in the coming years. Advances in lineage tracing and single-cell transcriptome analysis technologies can be readily adopted for use in this model organism, which will allow us to dissect the molecular identity of individual cells in the fly brain, as well as identify new Notch target genes involved in the complex interplay of genetic networks generating the cell type diversity of the brain.

A number of promising research directions are emerging in the study of *Drosophila* neuroblasts. The type II neuroblast lineages are an excellent comparative model for the study of mammalian neural stem cell lineages due to the presence of analogous transit-amplifying cell populations, which may be regulated by evolutionarily highly conserved mechanisms. In the future, it is critical to further understand how INPs undergo maturation and how they are prevented from dedifferentiation. The neuroepithelial stem cells in the larval optic lobe will continue to serve as an alternative good model to study the transition from SCD to ACD as well as tumourigenesis. Besides, a recent *Drosophila* study revealed that mutations in *l(3)mbt* cause brain tumour induction due to soma-to-germline transformation of the *l(3)mbt*-deficient cells [132]. In humans, cancer-testis genes, or cancer-germline genes, are aberrantly activated in various malignancies (reviewed in [133]). Studying these related genes in *Drosophila* are likely to provide important insight into the pathological mechanisms underlying the generation of soma-to-germline transformation-induced tumours.

The genes encoding the core proteins of the ACD apparatus and the asymmetrically localized polarity proteins and cell fate specification determinants are highly conserved in evolution. Therefore the findings obtained from studying *Drosophila* neuroblast self-renewal, cell fate specification and ACD regulation may be readily applicable and transferable for the unravelling of the mechanisms governing human neural stem cell biology and the pathological lesions underlying tumour formation derived from cancer stem cell populations. In this review, we present results from recent studies, which shows that the ACD apparatus and regulatory machinery required for polarity axis formation and control of ACD are operational in a number of neuroblastoma cell lines, and in human and rodent brain tumour specimens. Importantly, ACD seems to be compromised in brain tumours with a preponderance of SCD over ACD compared with wild-type tissue. This change in mode of cell division in cancer stem cells is accompanied with cell fate specification defects promoting a tumourigenic phenotype in the resulting progeny. Thus, these results encouragingly suggest that many of the molecular players and cellular processes identified in *Drosophila* neuroblasts are also actively involved in regulating human neural stem cell lineages.

From the perspective of mitigating hyper-proliferation in brain tumours it is quite an interesting and surprising observation that the treatment of neuroblastoma cells with a MYC inhibitor was shown to change the balance between ACD and SCD in favour of cells undergoing ACD [107]. This raises the hope that therapeutic agents may be developed to affect the balance between SCD and ACD in tumour-initiating cell populations and cancer stem cells in order to modulate cancer malignancy and improve clinical outcome. Of course, a detailed understanding of the molecular mechanisms regulating ACD would profoundly help us in our efforts to find candidate targets suitable for pharmaceutical intervention. In this respect, the *Drosophila* neuroblast model, the genetic amenability of the fly and the availability of large RNAi stock collections targeting most genes in the fly genome are going to help advance and accelerate the discovery of genes regulating ACD. This will further our understanding of the regulatory networks controlling neural stem cell homoeostasis and help elucidate the pathological series of changes underlying the transformation of neural stem cells to tumour-forming neural cancer stem cells.
